# Tunable Light Field Modulations with Chip- and Fiber-Compatible Monolithic Dielectric Metasurfaces

**DOI:** 10.3390/nano13010069

**Published:** 2022-12-23

**Authors:** Bobo Du, Yunfan Xu, Huimin Ding, Weitao Jiang, Lei Zhang, Yanpeng Zhang

**Affiliations:** 1 Key Laboratory of Physical Electronics and Devices of Ministry of Education and Shaanxi Key Laboratory of Information Photonic Technique, School of Electronic Science and Engineering, Xi’an Jiaotong University, Xi’an 710049, China; 2 State Key Laboratory for Manufacturing Systems Engineering, Xi’an Jiaotong University, Xi’an 710049, China

**Keywords:** light field modulations, monolithic metasurfaces, tunability, chip platform, fiber-optic platform

## Abstract

Metasurfaces with a high engineering degree of freedom are promising building blocks for applications in metalenses, beam deflectors, metaholograms, sensing, and many others. Though the fundamental and technological challenges, proposing tunable metasurfaces is still possible. Previous efforts in this field are mainly taken on designing sophisticated structures with active materials introduced. Here, we present a generic kind of monolithic dielectric metasurfaces for tunable light field modulations. Changes in the period number and surrounding refractive index enable discrete and continuous modulations of spatial light fields, respectively. We exemplify this concept in monolithic Lithium Niobate metasurfaces for tunable metalenses and beam deflectors. The utilization of monolithic dielectric materials facilitates the ready integration of the metasurfaces with both chip and optical fiber platforms. This concept is not limited by the availability of active materials or expensive and time-consuming fabrication techniques, which can be applied to any transparent dielectric materials and various optical platforms.

## 1. Introduction

Metasurfaces formed with artificial nanoscale features were introduced to tailor and control light’s fundamental properties [1,2]. Following the pioneering work, considerable efforts have been made to develop functional metasurfaces addressing various application needs [3,4]. Overall, various types of light manipulations have been demonstrated, such as amplitude tuning, wavefront modulations, and polarization controls. For amplitude tuning, the light intensity of the reflection, transmission, or absorption of metasurfaces is changed [5,6,7]. In wavefront modulations, the phase of a propagating beam is tailored for focusing, steering, or others [8,9]. Polarization controls are of essential importance in cases where beam formation, polarimetry, or optical trapping is required [10,11].

Benefitting from the continuous research on metasurfaces, tremendous new functionalities have seen their successes, including metalenses [12,13], beam steering [14,15,16,17], metaholograms [18], versatile polarization generations [19,20], nonlinear optics [14,21], and many others. Remarkably, the achievements in design strategies, active materials, and tuning mechanisms inspire the historical developments of tunable metasurfaces, which hold the potential of dynamic light manipulations [22,23,24,25]. Basically, there are two tunability strategies drawing the main attentions, including tailoring the properties of nanoscale features [22] and modifying the surrounding medium [26]. Since then, tunable metasurfaces have been the research hotspot laying the path to multifunctional devices.

Additionally, different from those metasurfaces with sophisticated structures [27,28], monolithic configurations with both the nanoscale features and the substrate is the same material that possesses intrinsic integration in optical circuits [29,30]. The realizations of monolithic metasurfaces are mainly attributed to simultaneous optimizations of shape, size, and orientation of subwavelength resonators, which engineer the wavefronts and enable the desirable optical responses [31,32,33]. More recently, emerging material has been explored for monolithic metasurfaces [34]. Nevertheless, the monolithic configurations suffer from weaker confinements of the electromagnetic field; therefore, there are larger difficulties in design. However, few tunable devices based on monolithic metasurfaces have been reported till now. In addition, extended configurations of these monolithic metasurfaces on fibers are pending for flexible application scenarios.

Lithium niobate (LiNbO_3_, LN) has been regarded as an alternative material in the photonics community owing to its commercial availability and multifunctional properties, including excellent visible and infrared transparency and large nonlinear coefficients [35]. In this work, we present tunable monolithic metasurfaces for focusing and beam deflectors. LN, as a representative dielectric material, is used. By varying the surrounding refractive index (SRI) continuously, the focal length of metalenses and the deflection angle of beam deflectors can be tuned accordingly. In practice, active materials, e.g., liquid crystal and phase-change materials, are the potential as the surrounding medium to enable the tunability of the continuously tunable metasurfaces with elaborate fabrications in the future [36,37]. Moreover, altering the nanopillar number in one supercell changes the slope of the phase gradient of a metalens so that the deflection angle of beam deflectors shifts discretely. The proof-of-concept of tunable monolithic metasurfaces is generic and applicable to any transparent dielectric materials. More interestingly, this monolithic configuration is readily compatible with various optical platforms, like chips and optical fibers. Within the fiber-integrated framework, the combination of metasurfaces with optical fibers benefits from two sides: one is the improved flexibility of metasurfaces in terms of practical applications, and the other is the expanded functionality of fiber devices. For instance, metasurfaces are attractive in scanning imaging, while flexible fiber-based metasurfaces allow the opportunity for internal tissue endoscopy in vivo environments around medical diagnosis. On the other hand, in practical scenarios, fiber devices are extremely essential, and the implementation of metasurfaces on fiber would constitute a crucial step forward in the multifunctionality of fiber devices, e.g., in-fiber collimator.

## 2. Materials and Methods

**Figure 1:**
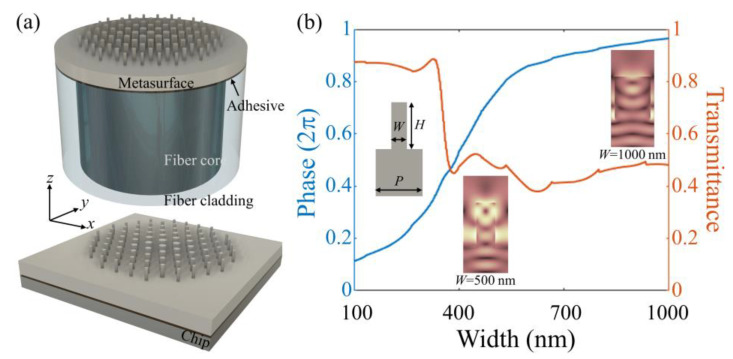
(**a**) Schematic of a monolithic dielectric metasurface on fiber (Top) and chip (Bottom) platform, respectively; (**b**) the phase delay and transmittance obtained via FDTD simulations of the LN nanopillar as a function of width *W*. The insets are the cell diagram and electric field profiles for *W* = 500 and 1000 nm from left to right, respectively.

The dielectric metasurfaces consist of nanopillars with rationally-arranged sizes, which are fabricated in monolithic materials, e.g., x-cut LN. Three-dimensional (3D) schematics of a metalense are present in Figure 1a. The structure of the metasurface is rotationally symmetric, allowing polarization-independent operations. The monolithic configuration is freestanding itself in an optical circuit, as well as compatible with optical fiber or chip platforms where the monolithic metasurfaces are integrated with one certain substrate via a UV-cured adhesive layer, as shown in the Top and Bottom of Figure 1a, respectively. The implementation can be achieved using an adopted transfer method that we used to fabricate plasmonic fiber tips [38]. During this process, the key challenge is probably the alignment issue. Indeed, taking advantage of the mature microscope and movement control techniques, the alignment could be well conducted [39]. As generally shown in Figure 1b, the nanopillar height *H* and array pitch *P* are optimized to provide 2π phase coverage through a range of nanopillar diameters. For simplification, not otherwise specified, two-dimensional (2D) simulation models in the x-z plane are built to investigate the performances of monolithic dielectric metasurfaces. The simulations were carried out using commercial software (Lumerical FDTD solution, Canada) with a laptop computer (Intel Core i7-10510U CPU, 16 GB RAM). In the finite difference time domain (FDTD) simulations, the spatial mesh grids are set as Δ*x* = Δ*y* = 10 nm. The source is set as a plane wave along the z-axis with a center wavelength of 1550 nm. The polarization of the incident beam is on the x-axis. For the convergence of the results, the temporal step and simulation time is set as 0.0233507 fs and 10,000 fs, respectively. At the wavelength of interest 1550 nm, the pitch *P* and the nanopillar height *H* are determined to be 1.2 μm, and the width of the LN nanopillar varies to achieve the desired phase delay. In this way, the waveguiding effect is responsible for phase accumulation, especially for larger nanopillar widths [40]. The corresponding phase delay and transmittance calculated by the FDTD method depend strongly on the nanopillar widths. Additionally, the simulated electric field profiles (standing-wave-like features) for LN nanopillar widths (500 nm and 1000 nm) verify the attributed waveguiding effect. The same anisotropic refractive index (*n_xx_* = *n_yy_* = *n*_o_ = 2.211, *n_zz_* = *n*_e_ = 2.138) is taken for LN [41] in our simulations. Note that in the phase and transmittance simulations, periodic boundary conditions are used.

According to the database in Figure 1b, the rational arrangements of LN pillars with corresponding widths at their positions offer specifically functional metasurfaces at the operation wavelength in transmission modes, such as metalens, beam deflectors, and many others. Low material losses and compressed scattering effects guarantee a fairly high transmission efficiency above 0.4 over the range of LN nanopillar sizes at the operation wavelength of 1550 nm.

## 3. Results and Discussions

### 3.1. Continuously Tunable Metalens

In the transmission mode, metalenses focus a collimated incident beam into a spot, i.e., the focal point. To accomplish this, the required profile of phase delay for a metalens needs to follow the hyperboloidal relation:

(1) $$\varphi_{d}\left(r,\lambda_{d}\right)=\varphi_{r}-\frac{2\pi }{\lambda_{d}}\left(\sqrt{r^{2}+f^{2}}-f\right),$$

where *φ*_r_ is the reference phase, *r* is the position of each dielectric element away from the center, *λ*_d_ is the operation wavelength in free space, and *f* is the focal length.

**Figure 2:**
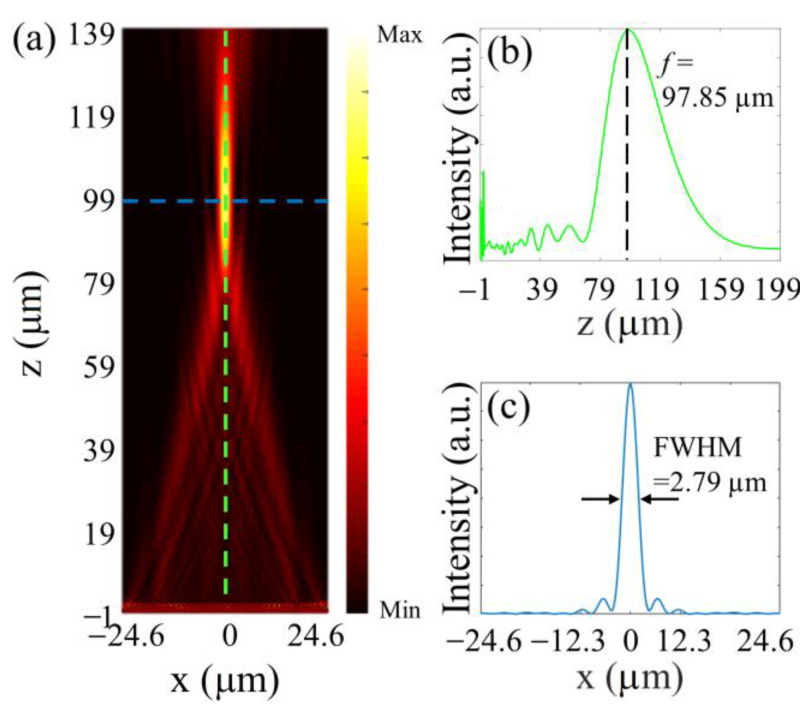
(**a**) Intensity profiles of the monolithic metalens along the propagation direction (*z*-axis) in the *x*-*z* planes. The longitudinal and horizontal cuts of the focal behavior are marked with green and blue dashed lines, as shown in (**b**,**c**), respectively.

To evaluate the focusing performance of the monolithic metalens, a metalens with a lateral size of 50 μm and designed focal length of 100 μm was simulated by FDTD. The size detail of the metalens can be found in Figure S1a (Supplementary Materials). The proposed metalens possesses a numerical aperture (NA) of 0.24. The calculated results of the power intensity of the metalens along the propagation direction (*z*-axis) are depicted in Figure 2a. It is clear that the transmitted beam propagates and focuses on a spot. The longitudinal and horizontal cuts of the focal behavior are marked with green and blue dashed lines, which are exhibited in Figure 2a,c, respectively. The calculated focal length is 97.85 μm agreeing well with the designed value. Well, the focal full-width at half-maximum (FWHM) of 2.79 μm exhibits the outstanding performance of the proposed monolithic metalens. Note also that in the FDTD calculation about metalenses, perfect matching layers (PMLs) boundary conditions are used. Additionally, the electric field profile near the metalens is shown in Figure S2 in the Supplementary Materials. Lobes are visible in the field distributions verifying the existence of waveguide modes along with Fabry–Perot effects responsible for the phase delay in the monolithic metasurfaces. Similar evidence could be found in previous work [29,34]. It should be noted that the current designs are for single-wavelength functionality; any wide-band operations would suffer from chromatic aberrations (Figure S3 in the Supplementary Materials), whereby achromatic metasurfaces are required. Moreover, monochromatic aberrations involve off-axis deviations of the incidence. Similar to common hyperbolic metalenses described by Equation 1, the designed monolithic metalens offers reliable focusing performances for paraxial cases [42]. More details can be found in the Supplementary Materials (Figure S4).

**Figure 3:**
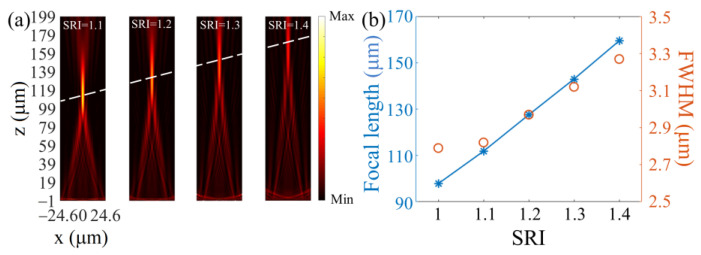
(**a**) Intensity profiles and (**b**) focal length and FWHM of the monolithic metalens as functions of the SRI.

Furthermore, we demonstrate the tunability of the monolithic metalens by adjusting the surrounding refractive index (SRI) where the metalens works. As summarized in Figure 3, with the SRI increasing from 1 to 1.4, the focal spot moves forward, and the focal length rises from 97.85 to 159.44 μm almost linearly. In addition, the focal spot slightly broadens horizontally, with the FWHM changing from 2.79 to 3.27 μm by 15%.

### 3.2. Discretely Tunable Beam Deflectors

A beam deflector with a phase gradient can shape the wavefront of the transmitted light and modulate the deflected light in one desired direction. The gradient phase profile of a beam deflector is described by the generalized Snell’s law as:

(2) $$n_{t}\sin\theta_{t}-n_{i}\sin\theta_{i}=\frac{\lambda_{d}}{2\pi }\frac{d\varphi_{d}}{dx},$$

where *θ*_t_ and *θ*_i_ are the incidence and deflection angles, respectively. *n*_t_ and *n*_i_ are the corresponding refractive indices of media at incidence and transmission sides, respectively. d*φ*_d_/d*x* is the phase gradient governing the beam deflection behaviors. For normal incidence (*θ*_i_ = 0) and air medium (*n*_t_ = 1), Equation (2) can be transformed into

(3) $$d\varphi_{d}\left(x,\lambda_{d},\theta_{t}\right)=\frac{2\pi }{\lambda_{d}}dx\sin\theta_{t},$$

**Figure 4:**
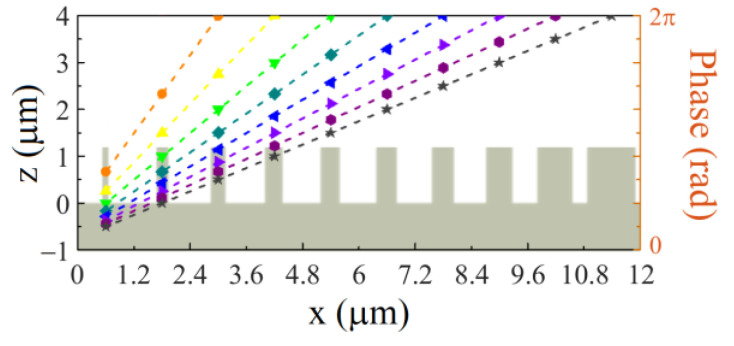
Schematic of the discretely tunable beam deflector. The dashed lines and dots represent the varied supercells with different numbers of nanopillars of metasurface deflectors.

Equation (3) presents freedom to engineer the deflection behaviors. For beam deflectors with 2π phase coverage, smaller lateral sizes lead to sharper phase gradients, therefore, larger deflection angles. As illustrated in Figure 4, to realize the phase gradient, a supercell consisting of a number of LN nanopillars with the widths rationally chosen is designed (Figure S1b in the Supplementary Materials). It is clear to see that different numbers of LN nanopillars with various widths donated by dashed lines and dots are arranged in one supercell to cover a full 2π phase delay range. By changing the nanopillar number from 3 to 10 within one supercell, the slopes of the phase gradient vary along with a full 2π phase accumulation over the supercell. According to Equation (3), the deflection angle is tuned discretely.

**Figure 5:**
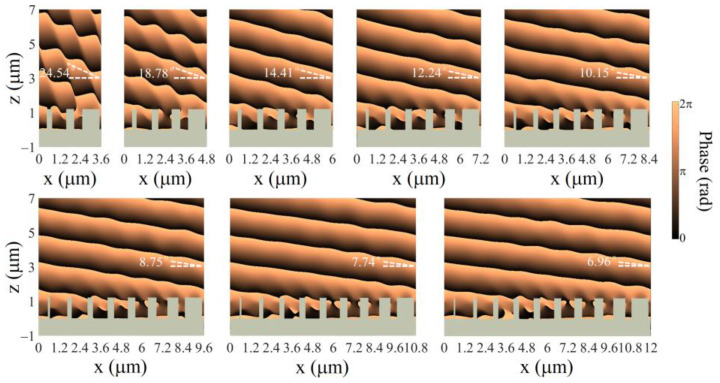
Simulated phase profiles of the deflected light beams through monolithic metasurface deflectors with varied numbers of LN nanopillars in one supercell.

Figure 5 summarizes the simulated phase profiles of the deflection behaviors of the monolithic beam deflectors with the supercell pitch *P*_s_ varying (*P*_s_ = *N* × *P*, *N* = 3, 4, …, 10). Note also that in the FDTD calculations here, plane-wave source and periodic boundary conditions are used. In order to visualize the deflection behaviors in the infinite metasurface deflectors, phase profiles of the deflected fields are obtained in simulations. As shown in Figure 5, when the incident light is illuminated from the bottom, each metasurface deflects the transmitted beam into one designed direction with high tunability. As the number of LN nanopillars forming the supercell increases from 3 to 10, the desired deflection angle through the monolithic metasurface deflector reduces from 25.50° to 7.42° discretely, while the actually simulated deflection angle changes from 24.54° to 6.96° correspondingly. The simulated and designed results show a good agreement with each other. The phase profiles clearly indicate the plane-wave features of the deflected light, suggesting the superior performances of the discretely tunable beam deflectors.

### 3.3. Continuously Tunable Beam Deflectors

**Figure 6:**
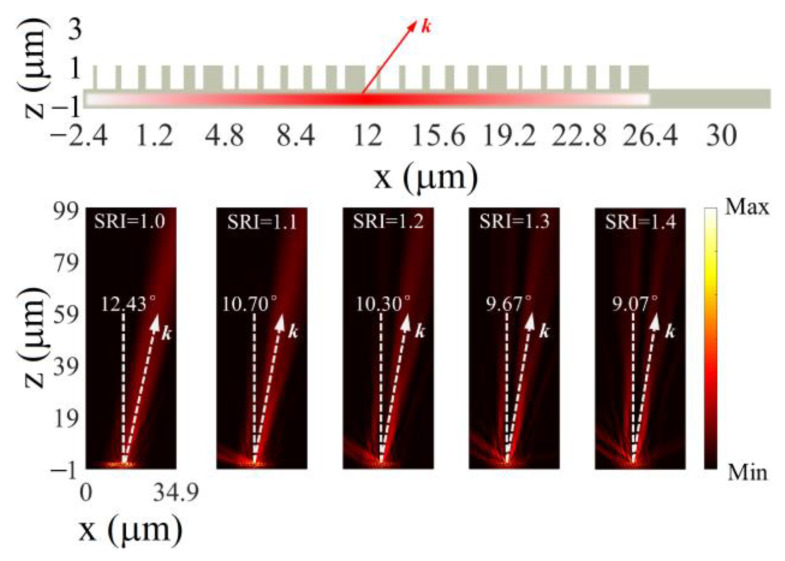
Deflection behaviors of a continuously tunable monolithic deflector. **Top**: configuration of the deflector. **Bottom**: simulated intensity profiles of the deflected beams under varied SRI.

Beyond monolithic metasurface metalenses, we demonstrate continuously tunable beam deflectors based on monolithic metasurfaces. As shown in Figure 6, four groups of supercells with six LN nanopillars are arranged to form the finite-size deflector. When a Gauss beam (indicated by the horizontal red bar) is injected into the deflector bottom, the transmitted light will deflect, and the wavevector *k* will orientate a certain direction. More interestingly, it is found that the deflection angle (wavevector orientation) strongly depends on the SRI. As the SRI rises from 1 to 1.4, the deflection angle decreases from 12.43° to 9.07° gradually and continuously. In practice, active materials, e.g., liquid crystal and phase-change materials, are available as the surrounding medium to enable the tunability of the continuously tunable beam deflectors. Continuous tunability offers a unique opportunity in high-performance light field modulations.

The above-designed monolithic metasurface deflectors consist of finite supercells of periodic LN nanopillars. Here, we present that the periodic lattice of LN nanopillars is not essential for the deflection function. By an elaborate arrangement of LN nanopillars offering a required phase difference throughout the metasurface size, the desired deflection angle is possible with a finite non-periodic lattice of LN nanopillars, as estimated by [43]

(4) $$\sin\theta_{t}=\frac{\lambda_{d}}{2\pi }\frac{d\varphi_{d}}{w},$$

where *w* is the whole lateral size of metasurfaces with nanopillars. Therefore, throughout the metasurface deflectors, the deflection angle is tunable by increasing the total phase difference or decreasing the metasurface size. Considering a monolithic LN metasurface deflector of 30 μm lateral size, a 25.91 rad phase difference would enable a 12.3° beam deflection.

**Figure 7:**
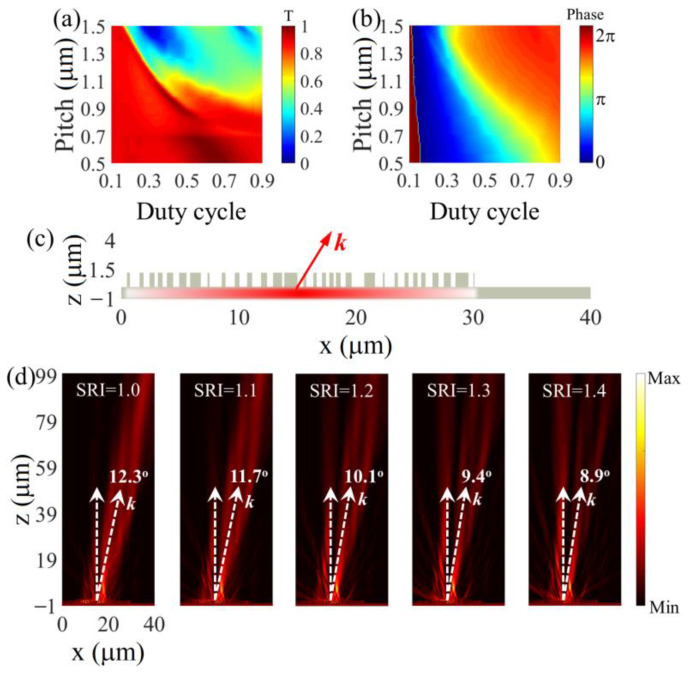
Dependences of (**a**) transmittance and (**b**) phase delay of LN nanopillar on lattice pitch and duty cycle, respectively. (**c**) Schematic and (**d**) SRI-tuned deflection behaviors of the finite non-periodic monolithic metasurface deflector.

As shown in Figure 7a,b, the lattice pitch and duty cycle of LN nanopillars are mapped to reveal the dependencies of transmittance and phase accumulation. By choosing the LN nanopillars with proper pitch and duty cycles (Figure S1c in the Supplementary Materials), a 25.91 rad phase difference is realized over a 30 μm metasurface for 12.3° beam deflection, as the diagram shows in Figure 7c. By altering the SRI from 1 to 1.4, a continuously tunable deflection behavior is observed with the finite non-periodic monolithic metasurface deflector. The deflection angle reduces from 12.3° in air (SRI = 1) to 8.9° in a medium of SRI = 1.4. The 3.4° tunability range is comparable with that of the finite periodic metasurface deflector present above.

**Figure 8:**
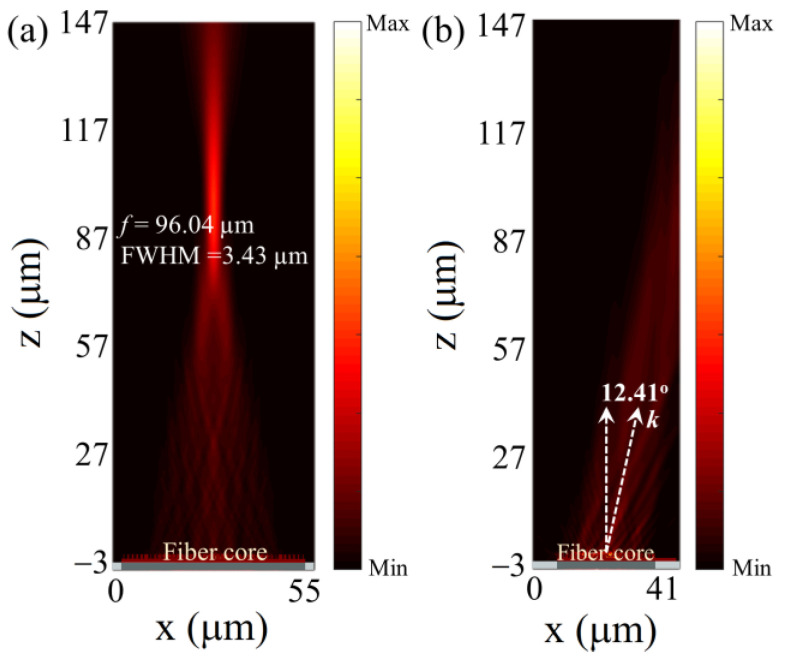
Performances of a monolithic metasurface (**a**) metalens and (**b**) beam deflector on multimode fiber tips, respectively. The diameter of the fiber core is as large as that of the correspondingly supported metasurface, i.e., (**a**) 50 μm and (**b**) 28.6 μm.

The performances of freestanding and chip-based monolithic metasurfaces are basically consistent since the illumination conditions and metasurface structures are the same despite the difference in supporting medium, i.e., monolithic metasurface body or extra materials. Indeed, the monolithic metasurfaces are compatible with optical fibers, which brings out those fiber meta-tips [44,45,46,47]. In terms of fiber-integrated monolithic metasurfaces, similar results can be achieved. As shown in Figure 8, a metalens and a beam deflector with the same parameters as above are integrated into a multimode fiber, respectively. The diameters of the fiber cores are the same large as those of the correspondingly supported metasurfaces (50 μm for metalens and 28.6 μm for beam deflector, respectively). The illumination source from fibers is a mode source with a fundamental mode in simulations which is different from the aforementioned plane wave or Gauss beam. It can be clearly seen from Figure 8a that the fiber-based metalens is able to focus the light beam into a spot, while the focal length and FWHM are 96.04 and 3.43 μm, respectively. These results are consistent with those based on a freestanding configuration in Figure 2. Figure 8b presents the deflection behavior of a monolithic metasurface deflector on a fiber tip. It is found that the fiber-based deflector offers a comparable deflection performance to that of the above continuously tunable beam deflectors (Figure 6, SRI = 1) with a deflection angle of 12.41°. The slight performance differences between the fiber-based monolithic metasurfaces compared with those of the above freestanding monolithic metasurfaces may be attributed to the illumination difference. Therefore, aiming at fiber-integrated circuits, the structures of the monolithic metasurfaces can be optimized specifically.

As extended contributions to the metasurface family of beam steering and varifocal lens, our monolithic dielectric metasurfaces take special advantages. As summarized in Table 1, our designs offer moderate modulation ranges both in beam steering and varifocal lens. Most importantly, the monolithic metasurfaces proposed in our work take the outstanding advantage of compatibility with varied optical platforms, i.e., chips and/or fibers. This flexibility would boost the variety of applications. Moreover, our designs exhibit good tunability continuities, excluding the discretely tunable deflectors through nanopillar number changing within one supercell.

## 4. Conclusions

This work presents a design route for fabricating tunable metasurfaces with monolithic dielectric materials. With metalenses and beam deflectors exemplified, discrete and continuous modulations of spatial light fields are realized through changes in nanopillar period number and refractive index surrounding the monolithic Lithium Niobate metasurfaces. This concept, with intrinsic integration to both chips and optical fibers, allows the efficient construction of tunable metasurfaces for plenty of applications taking advantage of a wide range of emerging dielectric materials.

## Figures and Tables

**Table 1:** Performance comparison among typical tunable metasurfaces.

Applications	Operation Wavelength	Tunability Range	Tunability Continuity	Compatibility	Ref.
Beam steering	0.65 μm	11°	No	Low	[48]
	0.672 THz	32°	No	Low	[49]
	1.522 μm	22°	No	Low	[50]
	5 THz	35.5°	Yes	Low	[51]
	0.917 μm	9.66°	No	Moderate	[52]
	1.55 μm	32°	No	Moderate	[53]
	0.124 μm	8.5°	No	High	[54]
	1.55 μm	27.58°	No	High	This work
	1.55 μm	3.4°	Yes	High	
Varifocal lens	1.522 μm	10 μm	No	Low	[50]
	0.633 μm	100 μm	Yes	Low	[55]
	0.532 μm	115 mm	Yes	Low	[56]
	1.55 μm	61.6 μm	Yes	High	This work

## Data Availability

The data presented in this study are available on request from the corresponding author.

## Supplementary Materials

The following supporting information can be downloaded at: https://www.mdpi.com/article/10.3390/nano13010069/s1, Figure S1: Nanopillar sizes of (a) the metalens, (b) the beam deflectors with varied numbers of nanopillars within one supercell, (c) the beam deflector based on non-periodic arrangement of nanopillars; Figure S2: Numerical result of the electric field profile of the metalens at wavelength of 1550 nm; Figure S3: Focusing performances of the monolithic metalens at different wavelengths (a) 1400 nm, (b) 1550 nm, and (c) 1600 nm; Figure S4: Optical aberrations of the monolithic metalens for paraxial cases with varied AOIs. (a) Simulated intensity profiles and (b) MTF.
