# Supplementary material for: Tunable Light Field Modulations with Chip- and Fiber-Compatible Monolithic Dielectric Metasurfaces
Source: Nanomaterials (Basel). 2022 Dec 23;13(1):69. doi: 10.3390/nano13010069 (PMC9823379; doi:10.3390/nano13010069)
Supplement: Supplementary file 1 [file nanomaterials-13-00069-s001.zip › nanomaterials-2071762-supplementary.pdf]

nanomaterials MDPI

Supplementary Materials

Tunable light field modulations with chip- and fiber-compatible monolithic dielectric metasurfaces

Bobo Du [1,*], Yunfan Xu [1], Huimin Ding [1], Weitao Jiang [2], Lei Zhang [1,*] and Yanpeng Zhang [1]

1 Key Laboratory of Physical Electronics and Devices of Ministry of Education and Shaanxi Key Laboratory of Information Photonic Technique, School of Electronic Science and Engineering, Xi’an Jiaotong University, Xi’an 710049, China; xuyunfan@stu.xjtu.edu.cn (Y.X.), dinghuimin@stu.xjtu.edu.cn (H.D.), ypzhang@mail.xjtu.edu.cn (Y.Z.)
2 State Key Laboratory for Manufacturing Systems Engineering, Xi'an Jiaotong University, Xi'an 710049, China; wtjiang@mail.xjtu.edu.cn
* Correspondence: bobo.du@xjtu.edu.cn (B.D.), eiezhanglei@xjtu.edu.cn (L.Z.)

Figure S1 shows the applied sizes of LN nanopillars for the proposed monolithic metasurfaces in our work. Figure S1a plots the size profile of metalens, while Figure S1b shows the size profiles for deflectors through the tailoring of LN nanopillar (NP) number within one supercell. The pitch and height both in Figure S1a and Figure S1b is 1.2 μm. For the non-periodic metasurface deflector, the pitch and duty cycle (Figure S1c) are selected to form the phase gradient of 25.91 rad over the 30 μm metasurface for 12.3° deflection. Figure R1(a), R1(b), and R1(c) is correlated to Figure 2, 4, and 7 in the main text, respectively.

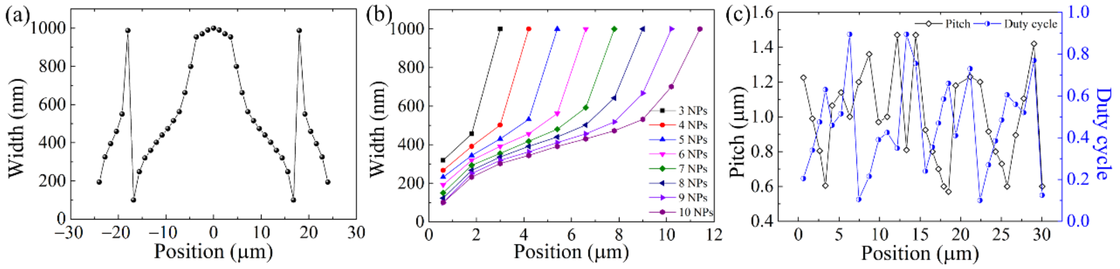

Figure S1. Nanopillar sizes of (a) the metalens, (b) the beam deflectors with varied numbers of nanopillars within one supercell, (c) the beam deflector based on non-periodic arrangement of nanopillars.

Figure S2 depicts a 2D simulation for the monolithic LN metalens in x-z plane at the wavelength of 1550 nm. It can be found that there exist lobes in vertical direction in the electric field distributions, suggesting the waveguide modes along with Fabry-Perot effects.

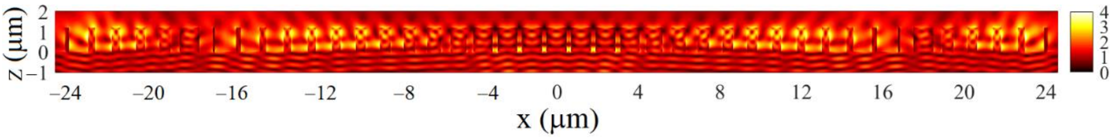

Figure S2. Numerical result of the electric field profile of the metalens at wavelength of 1550 nm.

Figure S3 presents the focusing performances of the metalens with the same parameters as that in Figure 2 in the main text supports different focal lengths of 105.89, 97.85, and 95.92 μm at wavelengths of 1.4, 1.55, and 1.6 μm. This is the evidence for the dispersion of light. However, in our work, we focus on the operations at the most popular telecommunication band of 1550 nm so that the dispersion is not involved.

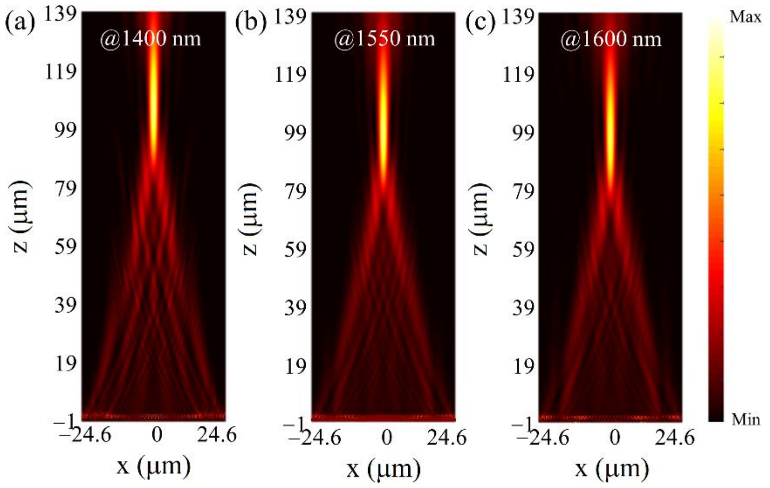

Figure S3. Focusing performances of the monolithic metalens at different wavelengths (a) 1400 nm, (b) 1550 nm, and (c) 1600 nm.

Figure S4 describes the impacts of angle of incidences (AOIs) on the focusing performances of the monolithic metalens in Figure 2 of the main text. The optical field is tightly focused at a spot with the same focal length for all cases (Figure S4a). The consistent performances are verified by the modulation transfer functions (MTFs) as well (Figure S4b). The MTF was obtained by taking the Fourier transform of the simulated focal plane intensity distributions along x-axis presented in Figure S4a. It is found that the designed monolithic metalens in our work offers reliable focusing characteristics at least for paraxial situations.

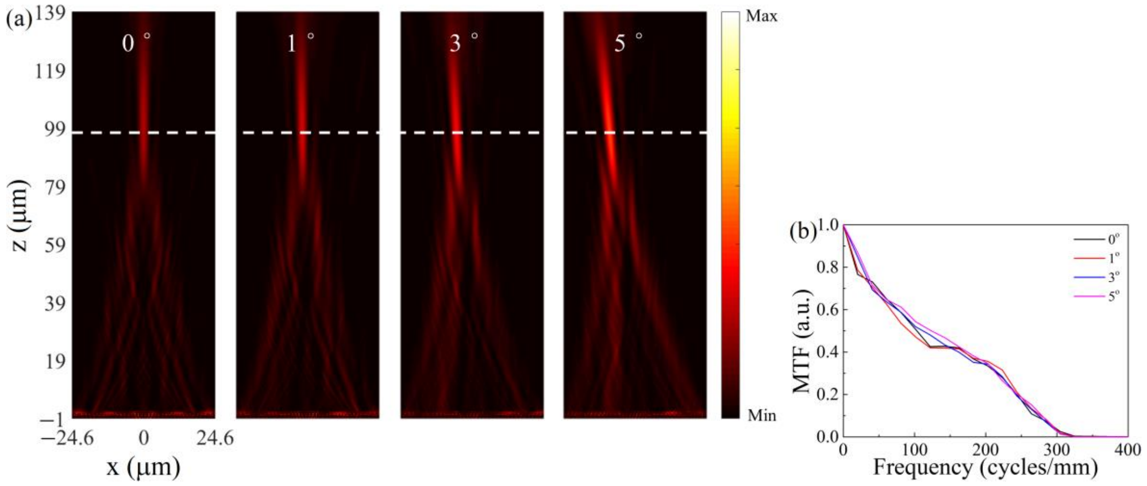

Figure S4. Optical aberrations of the monolithic metalens for paraxial cases with varied AOIs. (a) Simulated intensity profiles and (b) MTF.
